# Multi-domain semantic similarity in biomedical research

**DOI:** 10.1186/s12859-019-2810-9

**Published:** 2019-05-29

**Authors:** João D. Ferreira, Francisco M. Couto

**Affiliations:** LASIGE, Faculdade de Ciências, Universidade de Lisboa, Lisboa, Portugal

**Keywords:** Semantic similarity, Epidemiology, Multiple-domain, Prediction

## Abstract

**Background:**

Given the increasing amount of biomedical resources that are being annotated with concepts from more than one ontology and covering multiple domains of knowledge, it is important to devise mechanisms to compare these resources that take into account the various domains of annotation. For example, metabolic pathways are annotated with their enzymes and their metabolites, and thus similarity measures should compare them with respect to both of those domains simultaneously.

**Results:**

In this paper, we propose two approaches to lift existing single-ontology semantic similarity measures into multi-domain measures. The *aggregative* approach compares domains independently and averages the various similarity values into a final score. The *integrative* approach integrates all the relevant ontologies into a single one, calculating similarity in the resulting multi-domain ontology using the single-ontology measure.

**Conclusions:**

We evaluated the two approaches in a multidisciplinary epidemiology dataset by evaluating the capacity of the similarity measures to predict new annotations based on the existing ones. The results show a promising increase in performance of the multi-domain measures over the single-ontology ones in the vast majority of the cases. These results show that multi-domain measures outperform single-domain ones, and should be considered by the community as a starting point to study more efficient multi-domain semantic similarity measures.

## Background

Ontology-based semantic similarity uses the machine-readable definitions of concepts provided by ontologies to compare annotated entities based on their *meaning*. Contrast this with other similarity measures that use *structural* and/or *physical* properties of the entities: *e.g.* proteins have traditionally been compared based on their aminoacid sequence, chemical compounds on the graph representing their molecular structure, *etc*. While non-semantic measures are effective to a certain degree, they fail in some edge cases, such as proteins with similar functions having different sequences, or chemical compounds with similar molecular structure having disparate biological roles.

Semantic similarity between annotated biomedical resources has been a topic of research since Lord et al. [1] applied this technique to annotated proteins, as a search tool within a protein database. With the increase in the amount of biomedical domains being represented in formal ontologies, the desire to use ontologies to annotate biomedical entities increases, which resulted in multiple ontologies being used to that effect: metabolic pathways [2, 3], mathematical models of biological processes [4], functional tissue units [5], epidemiological resources [6], biomedical text and clinical notes [7], chemical toxicity [8] *etc*. These multidisciplinary entities, along with their multi-ontology annotations, can be regarded as biomedical digital resources that describe complex real-world phenomena.

Given the success of single-ontology semantic similarity measures in the past, for example, to assist text-mining [9–12], machine-learning [13–16], differential diagnosis [17], visualization [18, 19], *etc*., we argue that semantic similarity measures need to be developed to handle the multidisciplinarity of these types of resources; nevertheless, research in this field is still stalled in the single-domain world. For example, to compare metabolic pathways, Clemente et al. [20] used semantic similarity between its enzymes, and Grego et al. [21] used semantic similarity between its metabolites; a more accurate approach, however, would take into consideration both the enzymatic and chemical domains: the simultaneous use of both types of information should, in theory, provide a more accurate insight into what the pathways represent in the real world and, ultimately, contribute to a similarity measure more aligned with the scientific knowledge that surrounds the pathways.

However, to the best of our knowledge, this type of algorithm has yet to be fully studied within this community. Two recent papers have been presented that tackle them [22, 23]: Ning et al. [23] propose and evaluate semantic similarity of biomedical terms using four Chinese ontologies using path-based measure of similarity, and Cheng et al. [23] propose a gene-specific methodology to measure similarity between terms form different ontologies. However neither of those previous approaches is comparable to ours:

- Ning et al. [22] propose a way to aggregate semantic similarity calculated with various ontologies. While the work is in principle very similar to ours, they only use path-based measures of similarity, which are known to suffer from various drawbacks, particularly in the biomedical field (see [24]). Their aggregation approaches seem to be designed to overcome those limitation; our approaches, however, already integrate node-based measures, which take care of those limitations themselves.
- Cheng et al. [23] designed a way to compare concepts from different ontologies by exploring gene-related networks (protein-protein interaction networks, gene-regulation networks, etc.). Our work attempts to be more generic and works even if the domains do not have any connection with genes or genomics.

Instead of a new measure of semantic similarity designed from scratch to handle multidisciplinarity, we propose two approaches that can *lift* single-ontology measures into multi-domain measures. The “aggregative” approach compares each of the domains of relevance independently using existing single-ontology measures and then aggregates the several calculated values; the “integrative” approach integrates all the ontologies under the same common root and then applies single-ontology measures on it.

To assess the performance of the different approaches, we selected as case study a dataset of epidemiology resources, an inherently multidisciplinary field of research.

The results obtained with this dataset are meant to achieve two goals: (a) we show that the proposed approaches to the multi-domain similarity problem are effective, at least in comparison with the single-ontology counterpart; and (b) we hope to stimulate the community to think about the problematic of multidisciplinary similarity surrounding the ideas of knowledge representation and ontologies in the biomedical domain.

## Methods

Multi-ontology semantic similarity comes in two flavours: “single-domain” and “multi-domain”. “Single-domain multi-ontology” semantic similarity is a technique that takes into account multiple ontologies that try to represent the same domain of knowledge, *i.e.* the ontologies have common concepts that represent the same real-world ideas, for example two ontologies of anatomy. The existence of these various ontologies that represent the same domain can result from the ontologies offering complementary views of the reality. Some previous work has been carried out with respect to this type of semantic similarity [25–27]. Contrast this with “multi-domain multi-ontology” measures, which use ontologies representing different domains of reality. In this case, the ontologies are orthogonal, *i.e.* they represent different domains of reality, and thus rarely have concepts in common, and when they do, the overlapping concepts are very general. This type of measure is able to compare resources annotated with concepts from multiple domains of knowledge, as the biomedical entities mentioned above.

Notice that we are considering the multidisciplinarity of biomedical resources from the point of view of knowledge representation (KR): we propose a means to explore the ontology-provided definition of concepts to compare multidisciplinary entities annotated with concepts from more than one ontology (non-KR measures exist that are agnostic to the issue of multiple domains; *e.g.* Pederson et al. [28] compare concepts by comparing the textual neighbourhood of the concepts—the set of words that often appear *near* the concept in scientific literature).

Instead of creating a multi-domain measure from scratch, our methodology is to leverage on existing single-ontology measures, which have already been validated in a variety of scenarios, and *lift* them into multi-domain measures. As such, both the “aggregative” and “integrative” approaches take as input a single-ontology semantic similarity measure able to compare a set of concepts with another set of concepts (often called *groupwise* measures [24]).

**Fig. 1:**
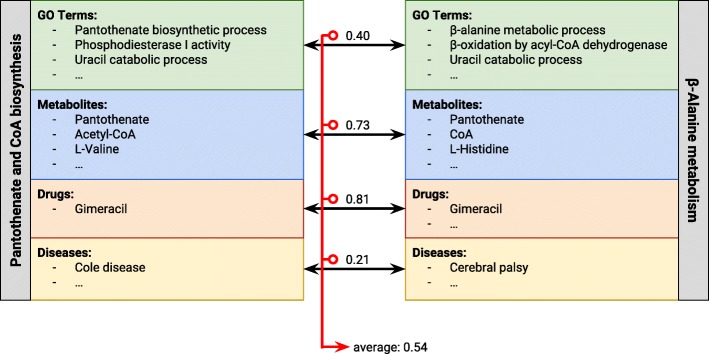
The aggregative approach. For each annotation domain in the entities being compared, the concepts in the first resource are compared with the concepts in the second. All the similarity values are aggregated into a final similarity score between, for example by using the average

The “aggregative” approach is depicted in Fig. 1. In this approach we independently compare each domain using a single-ontology measure, *i.e.* the concepts from one domain in the first resource are compared to the concepts from the same domain in the second resource. We do this for all the domains used to annotate the resources and aggregate these single-ontology results into a single value by using an aggregating function such as the raw average, where all domains weight the same, or the weighted average, where each domain is weighted proportionally to the number of concepts used to annotate the resources in that domain. Schliker & Albrecht [29] propose a similar methodology, where semantic similarity in the Gene Ontology (GO) is calculated independently for each of the three branches of this ontology, and then aggregated into a final similarity score. Ning et al. [22] also use similar methods to aggregate similarity in each ontology into a final multi-ontology value.

**Fig. 2:**
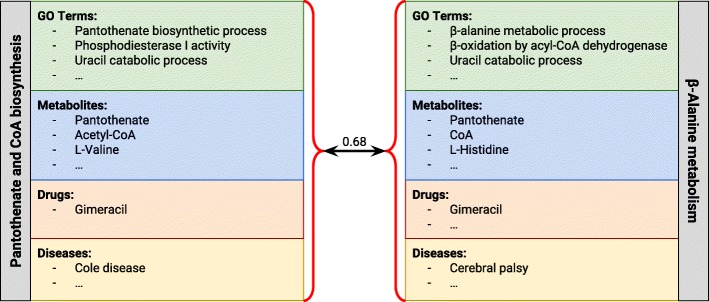
The integrative approach. All the concepts, irrespective of domain, are used to perform semantic similarity, which is done not with the individual ontologies but using a multi-domain ontology that consists of all the various ontologies merged under the same root. Only one similarity measure is used, resulting in a single final value

The “integrative” approach consists in merging the relevant domain-specific ontologies into a single multi-domain ontology. In case the ontologies share a common upper ontology (as is common in the biomedical domain, where reference ontologies are expected to be derived from the Basic Formal Ontology [30]), this merging means that concepts from different ontologies have now common superclasses, even though they are from different domains. In the absence of a shared upper ontology, this merging is done by creating a root concept that subsumes all the root concepts of all the ontologies. We then use the single-ontology measure on top of this multi-domain ontology (see Fig. 2).

The integrative approach has the advantage of being easy to implement and to straightforwardly enable the application of existing measures that have been proved useful in other endeavours. Additionally, it does not make use of arbitrary parameters for the domain weights. It also has the advantage that it inherently takes care of equivalences in multiple ontologies. For example, if several ontologies contain the concept “Cell” <GO:0005623>, the integrative approach automatically considers the concept as a single one; and as such the similarity between subclasses of this concept can make use of their common ancestor even if the subclasses come from different domains (*e.g.* both “Native cell” <CL:0000003> from the domain of cellular lines, and “Balancer cell” <CTENO:0000057> from the domain of Ctenophore anatomy). In this case, given the common ancestor, our measure is able to provide a similarity between concepts from the different ontologies greater than 0.

However, sometimes the ontologies are not as interoperable as expected. For example, the Foundational Model of Anatomy and the Cell Ontology contain a concept that represents “Cell”, and this approach does not allow the measure to be aware of the fact that both represent the same thing and are, therefore, equivalent classes. On the plus side, these collisions are rare, and their number is decreasing, as the biomedical informatics community strives to create their ontologies in the most orthogonal way, with as much re-usability of concepts as possible [31]. This community effort has the effect that ontologies do not contain different representation of the same real-world concept. Therefore, whenever a semantic resource refers to concepts from distinct domains, it must necessarily refer to concepts from different ontologies, which explains the need to annotate a resource with concepts from multiple ontologies.

In a multi-domain context, therefore, we can separate our measures of semantic similarity in four different settings:

- **Baseline** This is a collection of measures, each corresponding to the single-ontology measure carried out in one of the domains used to annotate the entities. These measures serve as a baseline to determine whether the multi-domain approaches outperform single-ontology ones.
- **Aggregative (raw)** All the single-ontology values obtained with the baseline setting are averaged with equal weights.
- **Aggregative (weighted)** This is the same as last setting, except that the average of the values obtained for each domain are weighted in proportion to the number of annotations in that domain.
- **Integrative** All the ontologies relevant for the similarity calculation are merged into one ontology and then the single-ontology measure is applied to it.

## Results

### Multi-domain case study

Epidemiology is an inherently multidisciplinary subject, relying on areas of knowledge as diverse as medicine, biology, statistics, sociology and geography [32]. Even under the scope of medicine and biology, epidemiology deals with chemistry concepts, diseases, symptoms, environmental conditions, methods of transmission, vaccines *etc*. A multi-domain semantic similarity measure would enhance information retrieval mechanisms on a repository of epidemiology data. To support a meaningful search functionality, the repository has to show to the user a set of resources similar to their query, which requires a means to compare resources based not only on one domain of interest (such as diseases), but on all the domains of annotations of the resources. It is conceivable, for example, to imagine a user in need of data related to “flu” in “Europe” with “fever” and “sneezing” symptoms. A search engine needs to be able to deal with these domains in order to properly return to the user the set of resources they are requesting, in an order that meaningfully reflects the their relevance to the query.

In fact, the multidisciplinarity of epidemiology has been previously explored and a network of epidemiology-related ontologies has been created, which contains ontologies that represent most of the epidemiology domains mentioned above [33]. The network has been developed within the scope of an European project that developed the Epidemic Marketplace, a repository of epidemiology information [6] that used it to assist users annotate their resources, using ontology concepts as metadata.

The full set of 204 resources were extracted from the Epidemic Marketplace, each corresponding to a scientific paper published in an epidemiology journal and annotated with concepts from the aforementioned network of ontologies.

Among the annotations for these resources, some use concepts from the NCIT (National Cancer Institute Thesaurus) and MeSH (Medical Subject Headings), which are on the less formal end of the ontology spectrum, *i.e.* they resemble *ad-hoc* vocabularies more than formal ontologies, where the relationships between class and subclass do not always reflect subsumption (for example, in MeSH, “Population” is classified under “Population Characteristics”, and in NCIT “Inactivity” under “Physical activity” but no true hypernymy exist in these cases). Additionally, they are used in this dataset mainly to provide non-biomedical-specific concepts, such as “Family characteristics”, which belong to the socio-economic sub-domain of epidemiology. For these reasons, these annotations were not included in our analysis.

**Fig. 3:**
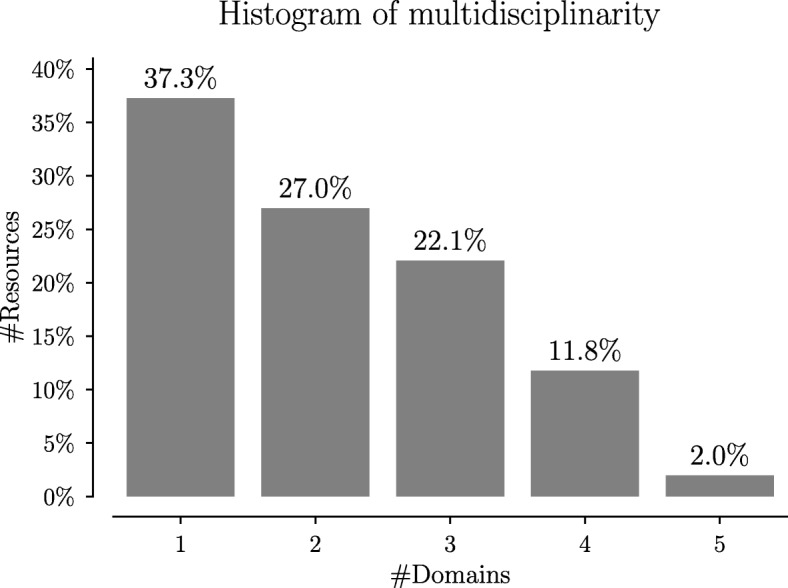
A histogram on the multidisciplinarity of the resources. The histogram shows how many of the 204 resources in the dataset have annotations in 1, 2, 3, 4 or 5 domains. While the most common value is 1 domain (37.3%), the majority of the resources (62.7%) have more than one domain of annotation

A summary of the relevant annotations for these resources is given in Table 1 and Fig. 3. The table shows that the resources are annotated with concepts from seven ontologies. These ontologies represent the domains of chemistry (CHEBI), diseases (DOID), environmental conditions (ENVO), phenotypic qualities (PATO), symptoms (SYMP), modes of disease transmission (TRANS) and vaccines (VO). In the table, **Coverage** is the fraction of resources that have at least one annotation in the specified domain, **Volume** is the average number of annotations from that domain within those resources, **Diversity** is the number of distinct concepts in that domain used in those annotations, and **Isolation** is the fraction of those resources that have annotations only in that domain. The figure shows that while a lot of resources are annotated with concepts from a single domain, the majority contain concepts from multiple domains. It also shows that the maximum number of domains is 5.

**Table 1:** Annotation statistics for the multi-domain resources extracted from the epidemic marketplace

Ontology	Statistics			
	Coverage	Volume	Diversity	Isolation
CHEBI	0.01	1.00	1	0.000
DOID	0.67	1.76	70	0.215
ENVO	0.24	1.00	9	0.020
PATO	0.01	1.00	1	0.000
SYMP	0.51	3.55	79	0.118
TRANS	0.48	1.00	9	0.010
VO	0.23	1.06	16	0.010

“Coverage” is the fraction of resources with annotations in each domain, “Volume” is the average number of annotations in these resources for that domain, “Diversity” is the number of distinct concepts of that domain used to annotate the resources, and “Isolation” is the fraction of resources that have only annotations in that domain

As can be seen from these results, each domain contributes with a partial description of the resources: there is a sparseness in the annotation profile, with many resources having annotations in only a few domains, and not always on the same domains. No domain covers the whole dataset, and most resources are annotated with more than one domain. Additionally, even though 37.3% of the resources are annotated in a single domain (*e.g.* 21.5% of the resources have DOID annotations only), it is not the same domain that covers those resources. As such, to compare the resources in this dataset using the classical single-ontology semantic similarity, it would be necessary to select one domain, which means that several of the resources would need to be left out of the analysis and that a high volume of information would be disregarded, as it belongs to some other domain. Multi-domain semantic similarity seems to be essential in this case study to enable a proper comparison of the resources.

### Evaluation

To assess the validity of semantic similarity in the case study dataset, we determined the degree to which it is possible to predict the DOID annotations from the other annotations. The rational behind this method is that performing a clinical diagnosis is equivalent to predicting the diseases based on other known factors (most notably symptoms) and is, therefore, one of the most important problems in biomedical informatics. In other words, we aim at predicting diseases that are related to a resource characterized by chemical compounds, environmental conditions, phenotype qualities, symptoms, modes of transmission and vaccines.

For this purpose, we used a multi-label machine learning algorithm, described by Zhang & Zhou [34]. This algorithm is known as ML-KNN, and uses a *k*-nearest neighbours (*k*-NN) approach to assign, to each resource, a set of DOID concepts. Using a *k*-NN-based algorithm is appropriate, since its performance highly depends on the performance of the similarity measure used to find the neighbours. The following steps describe ML-KNN:

1. Compare each resource *r* to the other resources, and determine the *k* most similar ones (this is the neighbourhood of *r*);
2. With these *k* resources, build a Bayesian model to calculate the probability that each DOID concept (from the set of all concepts in the DOID ontology) is also one of the annotations, based on the frequency with which each distinct possible concept appears in the *k* neighbours.
3. Compute a metric of performance based on the probabilities derived in the previous step. The ML-KNN paper suggests five different metrics, which we use here.

We executed these steps with each of the settings delineated in the previous section (the baselines, raw aggregative, weighted aggregative and integrative settings); we also ran the calculations using several different groupwise single-ontology measures (Resnik +BMA [35, 36], Lin +BMA [36, 37], simUI [38] and simGIC [36]).

**Fig. 4:**
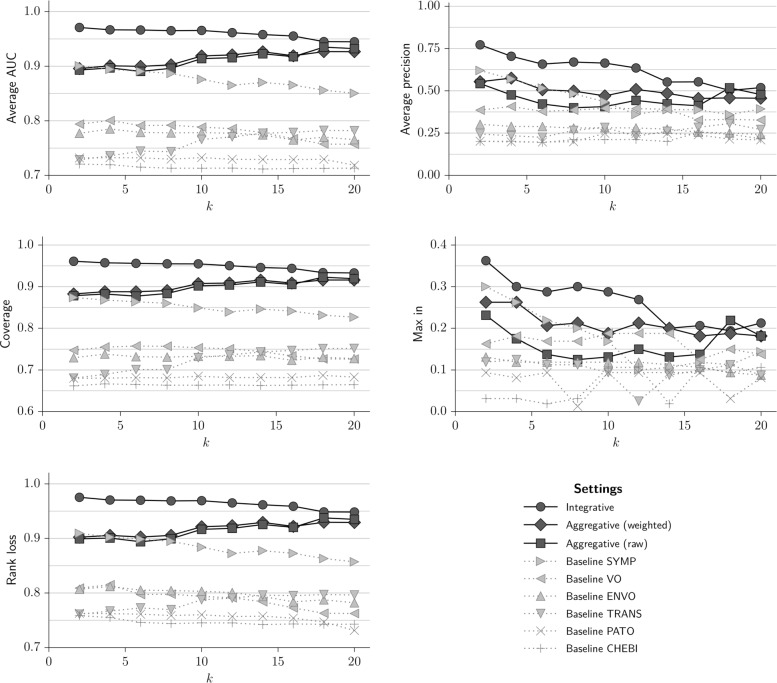
The performance of the various semantic similarity measures in the epidemiology dataset. The five graphs correspond to five different evaluation metrics calculated using the ML-KNN algorithm to predict DOID annotations for the resources in the dataset. Performance of single-ontology measures is presented as dotted grey lines, and performance of the multi-domain approaches is presented as black solid lines. The groupwise measure used in these results was Resnik +BMA

Figure 4 depicts the evaluation measure with respect to the several settings defined in the previous section using various values of *k*. These results were obtained using only the Resnik +BMA as the groupwise single-ontology similarity measure, because (i) the overall behaviour of the other groupwise measures does not differ significantly from the results that we are about to show, and (ii) this shows the best performance on this dataset. As can be observed, the integrative approach almost always outperforms the other settings, irrespective of evaluation metric and the value of *k*, which suggests that this measure is indeed superior to single-ontology measures in this dataset.

We can observe that the single-ontology measure performed on the “Symptoms” domain is the most successful baseline. This is justified by taking into consideration the annotation profile shown in Table 1. In fact, except for “Diseases”, this is the domain with the highest coverage, volume and diversity. Additionally, from the set of domains used to annotate these resources, symptoms are the most closely related to diseases. For small values of *k*, the performance of this baseline is either on par or above the performance of the aggregative approaches.

However, 88% of the resources have annotations to concepts from ontologies other than SYMP, which means that using only the “Symptoms” domain to measure similarity leaves out information; the results suggest that, in fact, incorporating other domains into the comparison algorithm increases the accuracy of the measure, as the evaluation metrics increase when we go from the SYMP baseline to the multi-domain measures.

It is interesting to notice the following overall trend: as *k* increases, the performance of the ML-KNN algorithm in the baseline settings decreases, especially for the SYMP baseline. This may be related to the fact that as we keep incorporating more and more neighbouring resources to predict DOID labels, we start including resources annotated with irrelevant symptoms, to the point where the extraneous information leads to a decrease in the algorithm’s performance. However, the performance for the TRANS baseline increases with *k*, and it appears that its incorporation in the multi-domain approaches helps keep the multi-domain performance either at a plateau or even to increase (for the aggregative measures). It is not immediately obvious why this difference in behaviour manifests in this dataset, and further studies would be needed to attribute a reason for this. For the moment, we believe that this can be explained by one of two reasons:

- There are only 9 distinct TRANS concepts used throughout the dataset, and only 48% of the resources have annotations in this domain. The increase suggests, then, that a similar mode of transmission is not directly indicative of similar diseases and only by probing further can this baseline be able to predict the correct DOID labels.
- The TRANS baseline has a generally low performance and the observed increase is not significant and can be mostly attributed to random chance.

Overall, the results consolidate the idea that taking into account multiple domains of annotation is relevant for obtaining useful similarity values.

### Predicting other labels

One way to summarize the results in the figure is by counting how many times the approaches with the *p*-highest performance are all multi-domain approaches, which we call *H*_*p*_. Since there are three different multi-domain approaches, it makes sense to compute *H*_*p*_ for *p*∈{1,2,3}. Also, since we performed an assessment with 5 distinct metrics and 10 different values of *k*, there is a total of 50 runs, which is the maximum value for *H*_*p*_.

Table 2 shows the values of *H*_1_, *H*_2_ and *H*_3_ obtained for the problem of predicting DOID labels (corresponding to the results shown in Fig. 3) as well as for the additional problems of predicting ENVO, TRANS, SYMP and VO labels. Given the low number of CHEBI and PATO annotations, we decided to remove them from this analysis.

**Table 2:** Summary of the performance of the similarity measures for distinct classification problems

Domain	*H*_1_	*H*_2_	*H*_3_
DOID	50	43	36
ENVO	50	47	44
SYMP	50	47	47
TRANS	50	45	36
VO	50	49	44

We performed the validation approach by predicting DOID labels, as well as labels for the ENVO, SYMP, TRANS and VO domains. This table displays the performance values *H*_*p*_ for those domains, with *p*∈{1,2,3}

This table shows that the top approach is always a multi-domain one. Also, the top three approaches are almost always the three multi-domain approaches as well. Despite this general trend, predicting DOID and TRANS labels seem to be the cases where *H*_*p*_ decreases the most. On the one hand, this may be related to the fact that predicting diseases is a non-trivial problem. On the other hand, as shown in Fig. 3, the “Max_in” metric seems unstable with respect to the change in *k*, which might help explain why these values are low in some of the classification problems.

## Discussion and conclusions

In this work, we demonstrate both the necessity and the feasibility of applying multi-domain semantic similarity measures in a dataset of epidemiology resources. Namely, we found that multi-domain semantic similarity measures can outperform single-ontology measures. This seems to be true especially when the annotations are sparsely distributed among the various domains. In these cases, all the present domains contribute, to some extent, to the final similarity score, increasing the accuracy of the measure. For example, if “SYMP” annotations are not enough to make a prediction, annotations to concepts from other ontologies can lead to an increase in accuracy.

The second fact extracted from the results is that the integrative approach almost always outperforms the single-ontology baselines and the other multi-domain approaches.

A third conclusion is that the “weighted aggregative” approach seems to be slightly more accurate than the “raw aggregative” approach (see the Max_in and Average precision metrics in Fig. 4). We conjecture that this happens because the weighted approach uses more information to calculate similarity. However, the two approaches present almost indistinguishable performance in the other evaluation metrics.

We would like to provide a few proposals for future work to address the limitations of our study.

First, we feel necessary to provide a few proposals for future work. First, while our results show the superiority of the integrative approach over the baselines, they were obtained by a validation method that cannot be directly applied to create new knowledge or to perform information retrieval. We would like to test these multi-domain approaches with actual data repository users (*e.g.* by validating whether a “Related resources” section actually provides related resources, or by testing whether the multi-domain semantic similarity can suggest data owners new annotations based on the ones already used to annotate a resource).

Other possible avenues to pursue include (i) trying new groupwise single-ontology measures in evaluating the behaviour of the aggregative and integrative approaches, and (ii) defining new aggregation methods for the aggregative approach (*e.g.* weighting the average on the information content of concepts rather than the amount of concepts in each domain).

Furthermore, ontologies are starting to make cross-references and to reuse concepts from one another. For example, some GO concepts make explicit references to CHEBI concepts: the formal definition of “carbohydrate binding” says that this is a subclass of the GO concept “binding” with an explicit relationship (“has_input”) to the CHEBI concept “carbohydrate”. Given that current single ontology measures are not able to exploit this inter-domain relationships directly, the “aggregative” and “integrative” approaches are also unable to use the cross-references. To solve this issue, we need to create new measures that properly explore such relationships. While such measures have still not been developed, we think that a measure proposed by us in the past could be a starting point to tackle this problem [39]. This measure builds a semantic neighbourhood of concepts based on the relationships between the concepts, and then compares two concepts based on the overlap of their neighbourhoods. By incorporating cross-references in the semantic neighbourhood, we can indeed include inter-domain knowledge in the multi-domain measure.

In conclusion, we present evidence to support the hypothesis that multi-domain semantic similarity is both necessary and feasible, and propose two approaches to lift single-ontology measures (which have been actively developed throughout the last two decades) into multi-domain measures. Therefore, this manuscript presents two main contributions: in multidisciplinary context we should not limit ourselves to single-ontology similarity, as that has negative implications on the overall performance of semantic similarity; and, by extension, we provide a baseline for future multi-domain measures.

## Abbreviations

BMA: Best match average
CHEBI: Chemical entities of biological interest
CL: Cell ontology
CTENO: Ctenophore ontology
DOID: Human disease ontology
ENVO: Environment ontology
GO: Gene ontology
*k*-NN: *k*-nearest neighbours
KR: Knowledge representation
MeSH: Medical subject headings
ML-KNN: Multi-label *k*-nearest neighbours
NCIT: National cancer institute thesaurus
PATO: Phenotype and trait ontology
SYMP: Symptoms ontology
TRANS: Modes of transmission ontology
VO: Vaccines ontology
